# Relationship Between Baroreflex and Cerebral Autoregulation in Patients With Cerebral Vasospasm After Aneurysmal Subarachnoid Hemorrhage

**DOI:** 10.3389/fneur.2021.740338

**Published:** 2022-01-12

**Authors:** Agnieszka Uryga, Nathalie Nasr, Magdalena Kasprowicz, Karol Budohoski, Marek Sykora, Peter Smielewski, Małgorzata Burzyńska, Marek Czosnyka

**Affiliations:** ^1^Department of Biomedical Engineering, Faculty of Fundamental Problems of Technology, Wroclaw University of Science and Technology, Wrocław, Poland; ^2^INSERM UMR 1297, Institute of Cardiovascular and Metabolic Diseases (I2MC), Toulouse, France; ^3^Department of Neurology, Toulouse University Hospital, Toulouse, France; ^4^Brain Physics Laboratory, Division of Neurosurgery, Department of Clinical Neurosciences, University of Cambridge, Cambridge, United Kingdom; ^5^Department of Neurology, St. John's Hospital, Vienna, Austria; ^6^Medical Faculty, Sigmund Freud University, Vienna, Austria; ^7^Department of Anaesthesiology and Intensive Care, Wroclaw Medical University, Wrocław, Poland; ^8^Institute of Electronic Systems, Faculty of Electronics and Information Technology, Warsaw University of Technology, Warsaw, Poland

**Keywords:** autonomic nervous system, subarachnoid hemorrhage, cerebral vasospasm, baroreflex, cerebral autoregulation, cerebrovascular regulation

## Abstract

**Introduction:** Common consequences following aneurysmal subarachnoid hemorrhage (aSAH) are cerebral vasospasm (CV), impaired cerebral autoregulation (CA), and disturbance in the autonomic nervous system, as indicated by lower baroreflex sensitivity (BRS). The compensatory interaction between BRS and CA has been shown in healthy volunteers and stable pathological conditions such as carotid atherosclerosis. The aim of this study was to investigate whether the inverse correlation between BRS and CA would be lost in patients after aSAH during vasospasm. A secondary objective was to analyze the time-trend of BRS after aSAH.

**Materials and Methods:** Retrospective analysis of prospectively collected data was performed at the Neuro-Critical Care Unit of Addenbrooke's Hospital (Cambridge, UK) between June 2010 and January 2012. The cerebral blood flow velocity (CBFV) was measured in the middle cerebral artery using transcranial Doppler ultrasonography (TCD). The arterial blood pressure (ABP) was monitored invasively through an arterial line. CA was quantified by the correlation coefficient (Mxa) between slow oscillations in ABP and CBFV. BRS was calculated using the sequential cross-correlation method using the ABP signal.

**Results:** A total of 73 patients with aSAH were included. The age [median (lower-upper quartile)] was 58 (50–67). WFNS scale was 2 (1–4) and the modified Fisher scale was 3 (1–3). In the total group, 31 patients (42%) had a CV and 42 (58%) had no CV. ABP and CBFV were higher in patients with CV during vasospasm compared to patients without CV (*p* = 0.001 and *p* < 0.001). There was no significant correlation between Mxa and BRS in patients with CV, neither during nor before vasospasm. In patients without CV, a significant, although moderate correlation was found between BRS and Mxa (r_S_ = 0.31; *p* = 0.040), with higher BRS being associated with worse CA. Multiple linear regression analysis showed a significant worsening of BRS after aSAH in patients with CV (*R*_p_ = −0.42; *p* < 0.001).

**Conclusions:** Inverse compensatory correlation between BRS and CA was lost in patients who developed CV after aSAH, both before and during vasospasm. The impact of these findings on the prognosis of aSAH should be investigated in larger studies.

## Introduction

Subarachnoid hemorrhage caused by the rupture of an aneurysm (aSAH), can lead to the neurological disability or death. The aSAH can cause molecular alterations (oxidative stress, inflammatory reaction) and loss of vascular integrity, leading to brain edema or delayed cerebral ischemia (1).

One of the most common complications following aSAH is cerebral vasospasm (CV), which occurs on average 4–7 days after ictus. Despite intensive research effort, CV following aSAH remains incompletely understood from a pathogenic perspective and does not solely explain the occurrence of delayed ischemic deficit (2). Several factors are potentially pathogenic: decreased nitric oxide (NO) availability, calcium-dependent vasoconstriction, free radicals, inflammation, dysfunctional endothelial neuronal apoptosis, or neurogenic factors (3). Impaired cerebral autoregulation (CA) might play an important role in the occurrence of delayed ischemic deficit in patients who developed CV after aSAH (4). Impairment of the cardiovascular autonomic nervous system in the acute phase of aSAH, as reflected by the low-baroreflex sensitivity (BRS), also appears to impact prognosis as it has been associated with adverse outcomes 3 months post aSAH (5). Furthermore, it has been shown that BRS was lower in non-survivors than in survivors. Moreover, patients with disturbed BRS had more extensive hemorrhage (6). Finally, both the impaired BRS and impaired CA have been associated with unfavorable prognoses. These two homeostatic mechanisms are inversely correlated in both the healthy volunteers and patients suffering from stable chronic diseases, such as carotid stenosis or occlusion (7). However, the way they interact in acute neurological diseases such as aSAH is not known. Short-term time trends of BRS in acute pathological events and more specifically in aSAH are not well-documented, in contrast to changes in BRS during medium- and long-term physiological conditions (8–10).

In this study, our primary objective was to assess CA and BRS in relation to the occurrence of CV. We hypothesized that the inverse correlation between BRS and CA previously found in physiology and in stable pathological disease could be lost in patients with aSAH and more specifically in patients with CV. A secondary objective of this study was to investigate the changes of BRS in the days following aSAH.

## Materials and Methods

### Study Design and Patient Management

**Figure 1:**
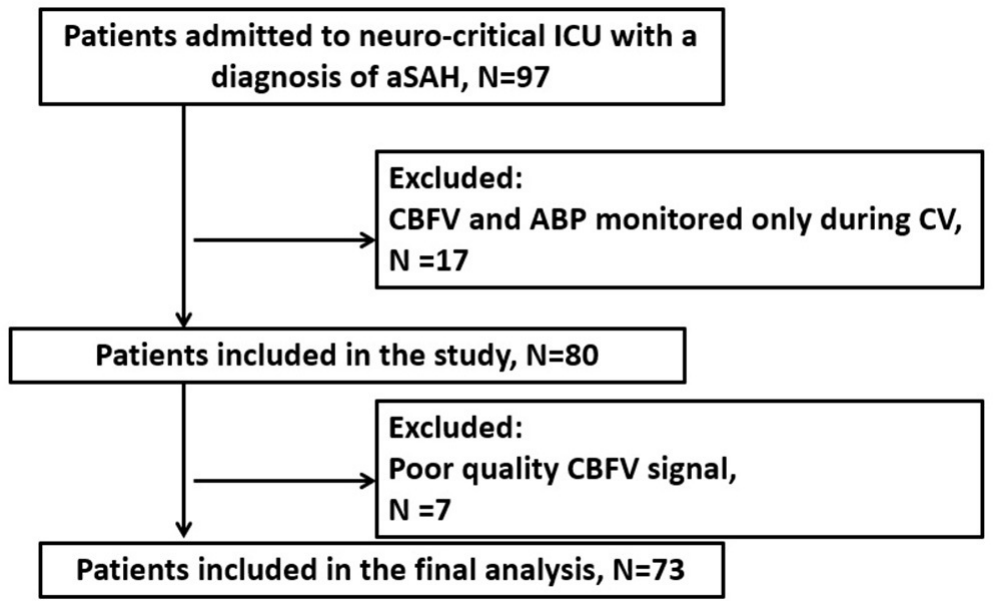
Flow diagram of the study. ABP, arterial blood pressure; CBFV, cerebral blood flow velocity; CV, cerebral vasospasm; aSAH, aneurysmal subarachnoid hemorrhage.

The study was a retrospective analysis of data prospectively collected at the neuro- critical care unit of Addenbrooke's Hospital (Cambridge, UK) between June 2010 and January 2012. A total of 97 patients suffering from aSAH were screened for compatibility with the study inclusion criteria: ≥18 years of age; admission to the hospital <5 days after aSAH with an intracerebral aneurysm likely to be the source of bleeding [as determined by computed tomography angiography or digital subtraction angiography (DSA)]; continuous multimodal monitoring. Patients were not enrolled in the study if they had autoregulation monitoring only during CV or if the quality of the signals was poor. The study was approved by the Research Ethics Committee of Addenbrooke's Hospital (Protocol 29 LREC: 97/291). All patients or their next-of-kin were required to sign written consent before inclusion. Seventy-three patients were enrolled for the study (flow chart depicted in Figure 1).

All patients were treated according to the guidelines applicable at the time of admission (11). Decisions concerning surgical clipping or endovascular embolization were made by the neurosurgical and interventional neuroradiology team. Conservative treatment was applied in a few patients with very severe and poor-grade aSAH. On admission, patients were assessed using the Glasgow Coma Scale (GCS). Neurologic state on admission was evaluated with the World Federation of Neurosurgical Societies (WFNS) grading system. The extent of hemorrhage was assessed using a modified Fisher (mFisher) scale and patients who had clinical or radiological signs of hydrocephalus were treated with external ventricular drainage (EVD), lumbar drainage, or serial lumbar punctures until the permanent diversion of cerebrospinal fluid was required. The treatment protocol included euvolemia, nimodipine, and cardiopulmonary support. Although “Triple-H” therapy was recommended at the time of admission, only induced hypertension was used. Delayed cerebral ischemia (DCI) was defined as focal neurological impairment (a two-point decrease in GCS) lasting for at least 1 h or cerebral infarction, which was not apparent immediately after aneurysm occlusion and was attributable to ischemia and not to other causes (12). Patients with diagnosed DCI underwent hemodynamic therapy with intravenous crystalloids administration to achieve central venous pressure between 8 and 12 mm Hg, as well as blood pressure support using individually titrated doses of vasopressors. The outcome was evaluated at discharge from the hospital and then after 3 months using the Modified Rankin Scale for Neurologic Disability (mRS). Scores of 0 to 2 on mRS were classified as good and scores of 3–6 on mRS were classified as poor outcomes.

### Signal Monitoring

Arterial blood pressure (ABP) was measured invasively in the radial artery using a pressure transducer and a pressure monitoring kit (Baxter Healthcare, Deerfield, IL, USA). Bilateral cerebral blood flow velocity (CBFV) in the middle cerebral artery (MCA) was monitored with transcranial Doppler ultrasonography (TCD) (DopplerBox; DWL Compumedics, Singen, Germany) using a head positioning device (Lam Rack, DWL Compumedics) through the temporal acoustic window. Data were acquired at a frequency of 200 Hz using an Intensive Care Monitor System (ICM+; Cambridge Enterprise Ltd, Cambridge, UK), and digitized using an analog-to-digital converter. All artifacts were identified manually or by custom-written algorithms. ABP and CBFV were measured in all patients.

**Figure 2:**
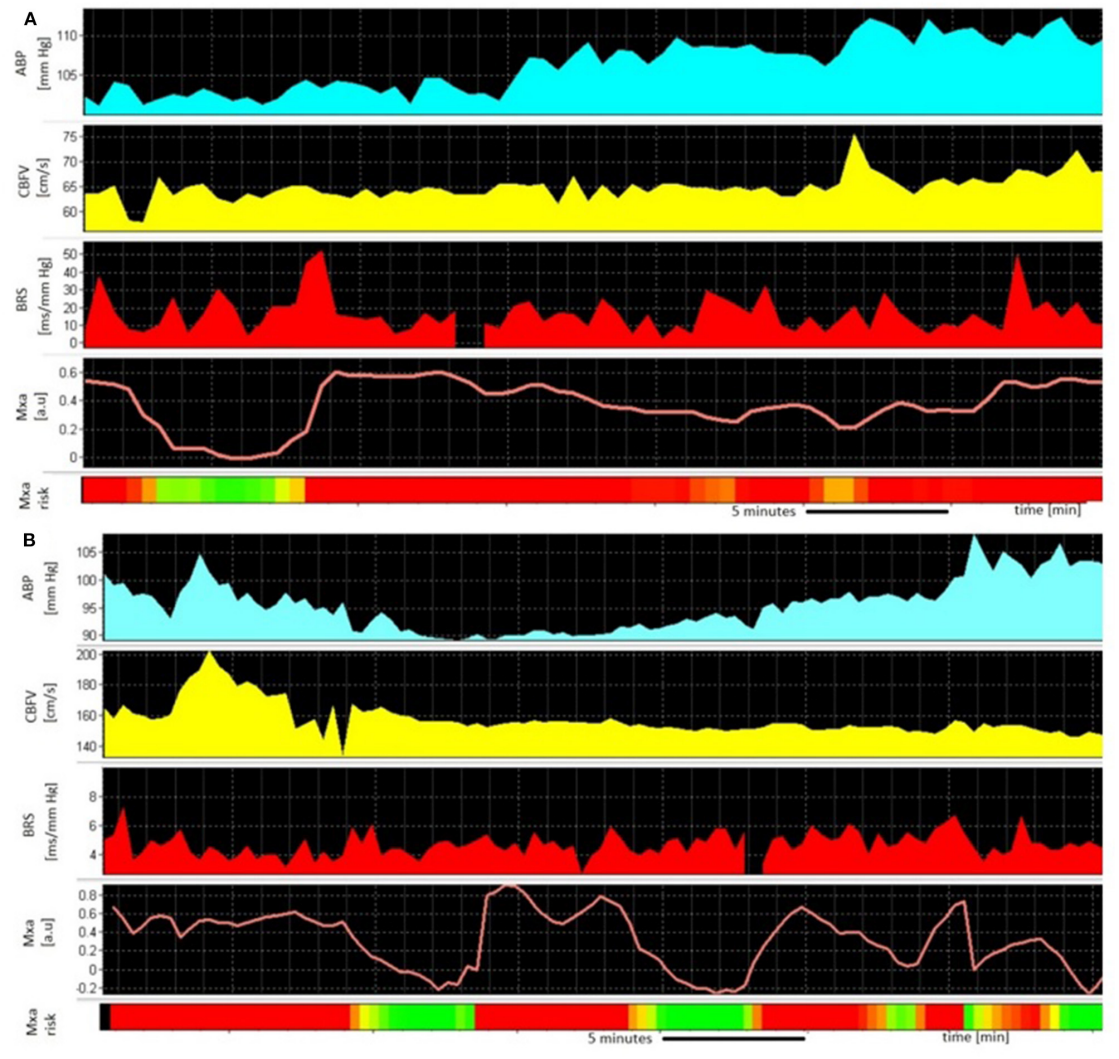
An example of the monitoring of **(A)** 45-year old man without cerebral vasospasm (CV) 12 days after aneurysmal subarachnoid hemorrhage (aSAH) from a ruptured aneurysm in the posterior cerebral artery (PCA). The high values of Mxa [worse cerebral autoregulation (CA), red panel] are counterbalanced with efficient, high-cardiac baroreflex sensitivity (BRS); **(B)** 67-year old woman during CV 8 days after aSAH from a ruptured aneurysm in the anterior communicating artery (ACom). The high values of Mxa (worse CA, red panel) occur in parallel with disrupted, low BRS; ABP, arterial blood pressure; CBFV, cerebral blood flow velocity; BRS, baroreflex sensitivity; Mxa, mean velocity index; the Mxa above 0.3 is interpreted as pathological.

Multimodal signal monitoring was started during the first days after admission. All patients had TCD performed every 1–2 days by a single investigator (BK). CBFV was monitored for about one hour; the minimum time was a half-hour and the maximum of one and a half hour. The median ± interquartile range (IQR) of the time of the start of the TCD recordings was 3 ± 3 days. The median ± IQR of the recordings per patient was 4 ± 3 (min-max: 1–11). There was one recording per day. The medians and IQRs of the observation time were: 11 days ± 5 days (min-max: 6–26 days) in the CV group and 7 days ± 5 days (min-max: 1–15 days) in the non-CV group, respectively. Illustrative time trends of the monitored signals are presented in Figure 2.

### Cerebral Vasospasm

Cerebral vasospasm was defined using TCD as mean CBFV in the MCA above 120 cm/s with a concomitant Lindegaard ratio (LR) above 3.0, where LR = CBFV_MCA_/CBFV_ICA_. When DSA was performed CV was defined as 25% narrowing of cerebral arteries on DSA irrespective of TCD findings. Routine postoperative CT and DSA were not performed. The side on which CV was diagnosed at the time of monitoring was labeled as the ipsilateral side. In patients in whom bilateral vasospasm was diagnosed, an average of parameters on both sides was used and labeled as the ipsilateral side. In patients in whom no vasospasm was diagnosed, the aneurysm side was used as the ipsilateral side. In patients with midline aneurysms but without diagnosed vasospasm, an average of parameters on both sides was used as the ipsilateral side. CBFV and CA were assessed on the ipsilateral side.

### Cerebral Autoregulation

CA was assessed from slow-wave oscillations of ABP and CBFV. The mean velocity index (Mxa) was calculated as the Pearson linear correlation coefficient between these signals (averaged over 10-s intervals) in a 5-min moving average window updated every 10 s. Values of Mxa >0.3 reflect impaired CA (13).

### Baroreflex Sensitivity

The BRS was calculated using the sequential cross-correlation method (also called x-BRS), published by Westerhof (14), which was embedded in ICM+. ABP systolic peaks were used to obtain RR interval time series. BRS was estimated as the slope of the regression line between the segments of systolic ABP (the input signal) and the RR interval (the output signal). BRS calculations were performed using a moving 10-s window along with mean values of the other vital signs variables included in the analysis. The BRS algorithm required the systolic ABP and RR time series to be incrementally shifted with respect to each other in search of the highest value of cross-correlation. Thus, the total window length used in each BRS calculation was extended to 17 s (7). BRS was returned when the *p*-value of the correlation coefficient was <0.01 and no ectopic beats were detected (15).

### Statistics

The normality distribution of the data was assessed using the Kruskal–Wallis test with the Lilliefors correction. Non–parametric tests were applied because the normality condition was not met for most of the analyzed parameters. The differences in median values were tested using the Mann–Whitney *U*-test. For non-numeric data, the Pearson CHI^2^ test (Fisher exact test/The Fisher-Freeman-Halton test) was used. McNemar's test was used to assess the increase in subjects with impaired CA during CV in comparison with the period before CV. Differences in median values before and during CV were compared using the Wilcoxon signed-rank test. Median values of CA and BRS were calculated using the entire time period in patients without CV, and using separately the time periods before and during vasospasm in patients with CV. Correlation analysis was performed using Spearman's rank test. The relationships between elapsed time (days) and physiological parameters were calculated using multiple linear regression analyses, with subjects treated as categorical factors using dummy variables (with respect to the inter– subject variability) and a partial coefficient (R_p_) between analyzed variables as recommended by Bland and Altman (16, 17). Results are presented as medians ± interquartile ranges or median (25–75th percentile) if not otherwise specified. The level of significance was set at 0.05. Statistical analysis was performed using STATISTICA version 13 (Tibco, Palo Alto, CA, USA).

## Results

### Baseline Characteristics

The group consisted of 24 (33%) men and 49 (67%) women, with a median age of 58 (50–67) years. Clinical data for the total group are presented in Table 1. Twenty-six patients (36%) underwent coiling and 51 (70%) underwent surgical clipping. In 5 patients (7%) conservative treatment was applied due to their severe condition. In this group, 3 patients died (60%), and these deaths were 50% of all deaths in this aSAH group. The 2 remaining patients were discharged from the hospital with good outcomes according to the evaluation of the mRS scale. Median WFNS scale was 2 (1–4) and median mFisher scale was 3 (1–3). CV was observed in 31 patients (42%) and occurred on day 8 ± 3. Mechanical ventilation was applied in 24 (33%) patients: 12 (39%) in the CV group and 12 (28%) in the non-CV group. Vasopressor agents were used in 26 (36%) patients: 18 (58%) in the CV group and 8 (19%) in the non-CV group.

**Table 1:** Patient characteristics.

	**All patients *n* = 73**	**CV *n* = 31**	**Non–CV *n* = 42**	***p***
Female, *n* (%)	49 (67%)	21 (64%)	28 (67%)	0.922^b^
Age (years), median (IQR)	58 (50–67)	55 (48–63)	62 (53–71)	**0.009** ^**a**^
GCS, median (IQR)	14 (11-15)	12 (11-15)	14 (13-15)	0.372^a^
mFisher, median (IQR)	3 (1-3)	3 (3)	3 (1-3)	0.415^a^
1, *n* (%)	19 (26%)	4 (13%)	15 (36%)	**0.030** ^b^
2, *n* (%)	4 (6%)	2 (6%)	2 (4%)	
3, *n* (%)	36 (49%)	21 (68%)	15 (36%)	
4, *n* (%)	14 (19%)	4 (13%)	10 (24%)	
WFNS, median (IQR)	2 (1-4)	2 (1-4)	2 (1-3)	0.499^a^
1, *n* (%)	26 (36%)	10 (32%)	16 (38%)	0.772^b^
2, *n* (%)	26 (32%)	10 (32%)	14 (33%)	
3, *n* (%)	4 (6%)	1 (3%)	3 (7%)	
4, *n* (%)	16 (22%)	9 (30%)	7 (17%)	
5, *n* (%)	3 (4%)	1 (3%)	2 (4%)	
**Treatments** ^**#**^				
Endovascular coiling, *n* (%)	26 (36%)	8 (26%)	18 (43%)	0.133^b^
Surgical clipping, *n* (%)	51 (70%)	25 (81%)	26 (62%)	0.085^b^
Conservative treatment, *n* (%)	5 (7%)	0 (0%)	5 (12%)	0.068^b^
Mechanical ventilation, *n* (%)	24 (33%)	12 (39%)	12 (28%)	0.327^b^
Vasopressor agents, *n* (%)	26 (36%)	18 (58%)	8 (19%)	**0.006** ^b^
**Complications**				
Hydrocephalus, *n* (%)	37 (51%)	18 (58%)	19 (45%)	0.279^b^
EVD, *n* (%)	24 (33%)	13 (42%)	11 (26%)	0.157^b^
DCI, *n* (%)	22 (30%)	19 (61%)	3 (7%)	**<0.001** ^b^
Rebleeding, *n* (%)	6 (8%)	1 (3%)	5 (12%)	0.227^b^
**Outcome**				
Deaths, *n* (%)	6 (8%)	2 (6%)	4 (10%)	0.491^b^
mRS at discharge, median (IQR)	2 (1-3)	2 (1-3)	2 (1-4)	0.648^a^
0–2, *n* (%)	46 (63%)	17 (55%)	29 (69%)	0.214^b^
3–6, *n* (%)	27 (37%)	14 (45%)	13 (31%)	
3 months mRS, median (IQR)	1 (0–2)	1 (0–2)	1 (0–2)	0.799^a^
0–2, *n* (%)	60 (82%)	35 (83%)	25 (81%)	0.767^b^
3–6, *n* (%)	13 (18%)	7 (17%)	6 (19%)	

*p-values marked with bold indicate statistically significant differences between the groups. CV, cerebral vasospasm; EVD, external ventricular drain; DCI, delayed cerebral ischemia; mRS, modified rankin scale; a–U mann-whitney test, b–Chi-squared test, #-More than one procedure per patient allowed*.

### Clinical Data in the CV and Non-CV Groups

A comparison of clinical data for the CV group and the non-CV group is presented in Table 1. There were no significant differences between the CV and the non-CV group for the following parameters: sex, WFNS, and GCS scales. There were no significant differences between the CV and the non-CV group concerning treatment: the proportion of patients with mechanical ventilation, coiling, or clipping did not differ significantly between the two groups. DCI occurred more frequently in patients with CV than without CV: 61% vs. 7%, *p* < 0.001. Patients with CV had more extended hemorrhage (mFisher 4–5): 81% in the CV group vs. 61% in the non-CV group, *p* = 0.030. Patients with CV were significantly younger compared to patients without CV: 55 (43–63) vs. 62 (53–71), *p* = 0.009. There were no significant differences in the outcome at discharge or after 3 months, nor in the death rate between the CV and the non-CV group.

### Physiological Metrics in the CV and Non-CV Groups

The results of the comparison of ABP, HR, and CBFV between the CV and non-CV groups are presented in Table 2. There was no significant difference in the physiological metrics between patients with CV in the period before vasospasm and averaged values of these parameters in patients without CV, except for a higher value of CBFV (*p* = 0.005, Table 2). In the CV group during vasospasm patients had significantly higher ABP (*p* = 0.001) and CBFV (*p* < 0.001) than averaged values of these parameters in patients without CV (Table 2). In the CV group during vasospasm, we observed a substantial increase in the following parameters compared with the period before vasospasm: ABP (*p* = 0.025), HR (*p* = 0.001), and CBFV (*p* < 0.001, Table 2).

**Table 2:** Physiological metrics and cerebral autoregulation in patients with and without cerebral vasospasm (CV).

**Parameter**	**No-CV *n* = 42**	**CV Before *n* = 31**	**CV During *n* = 31**	p*	***p^**#**^***	***p^†^***
ABP (mm Hg)	89.49 (90.51–80.87)	93.74 (82.91–106.44)	106.50 (89.70–116.65)	0.145	**0.001**	**0.025**
HR (bpm)	71.99 (64,03–76.19)	66.41 (61.12–81.15)	75.12 (63.76–86.92)	0.425	0.281	**0.001**
CBFV (cm/s)	65.33 (48.63–76.33)	76.65 (63.31–96.89)	143.20 (122.99–154.71)	**0.005**	**<0.001**	**<0.001**
Mxa ipsilateral (a.u.)	0.37 (0.30–0.46)	0.37 (0.28–0.42)	0.43 (0.36–0.52)	0.691	**0.039**	**0.002**
Mxa ipsilateral >0.3 [*n* (%)]	31 (74%)	21 (68%)	28 (90%)	0.571	0.068	**0.001**
BRS (ms/mm Hg)	10.92 (7.53–15.93)	13.73 (8.41–20.50)	9.53 (5.95–12.20)	0.130	0.244	**<0.001**

Data are presented as median (lower quartile–upper quartile). p-values marked with bold indicate statistically significant differences between the groups. Following comparisons are presented for patients: non-CV vs. before CV* (U Mann-Whitney test), non-CV vs. during CV^#^ (U Mann-Whitney test), before CV vs. during CV^†^ (Wilcoxon signed rank test); ABP, arterial blood pressure; HR, heart rate; CBFV, cerebral blood flow velocity; Mxa, mean velocity index of cerebral autoregulation; BRS, baroreflex sensitivity.

### Baroreflex Sensitivity and Cerebral Autoregulation in the CV and Non-CV Groups

There was no significant difference in BRS between patients without CV and patients with CV, neither in the period before vasospasm nor during vasospasm (Table 2). In the patients with CV, BRS decreased substantially during vasospasm compared with the period before vasospasm: 9.53 (5.95–12.20) (ms/mm Hg) vs. 13.73 (8.41–20.50) (ms/mm Hg), *p* < 0.001.

The CA was impaired in 74% of patients without CV. In the CV group CA was significantly worse during vasospasm than before vasospasm [Mxa: 0.43 (0.36–0.52) vs. 0.37 (0.28–0.42), *p* = 0.002]. Moreover, in the CV group the proportion of patients with impaired CA increased from 68% before vasospasm to almost 90% during vasospasm. According to McNemar's test, CV was significantly associated with worse CA (*p* = 0.001).

### Relationship Between Cerebral Autoregulation and Baroreflex Sensitivity in the CV and Non-CV Groups

**Figure 3:**
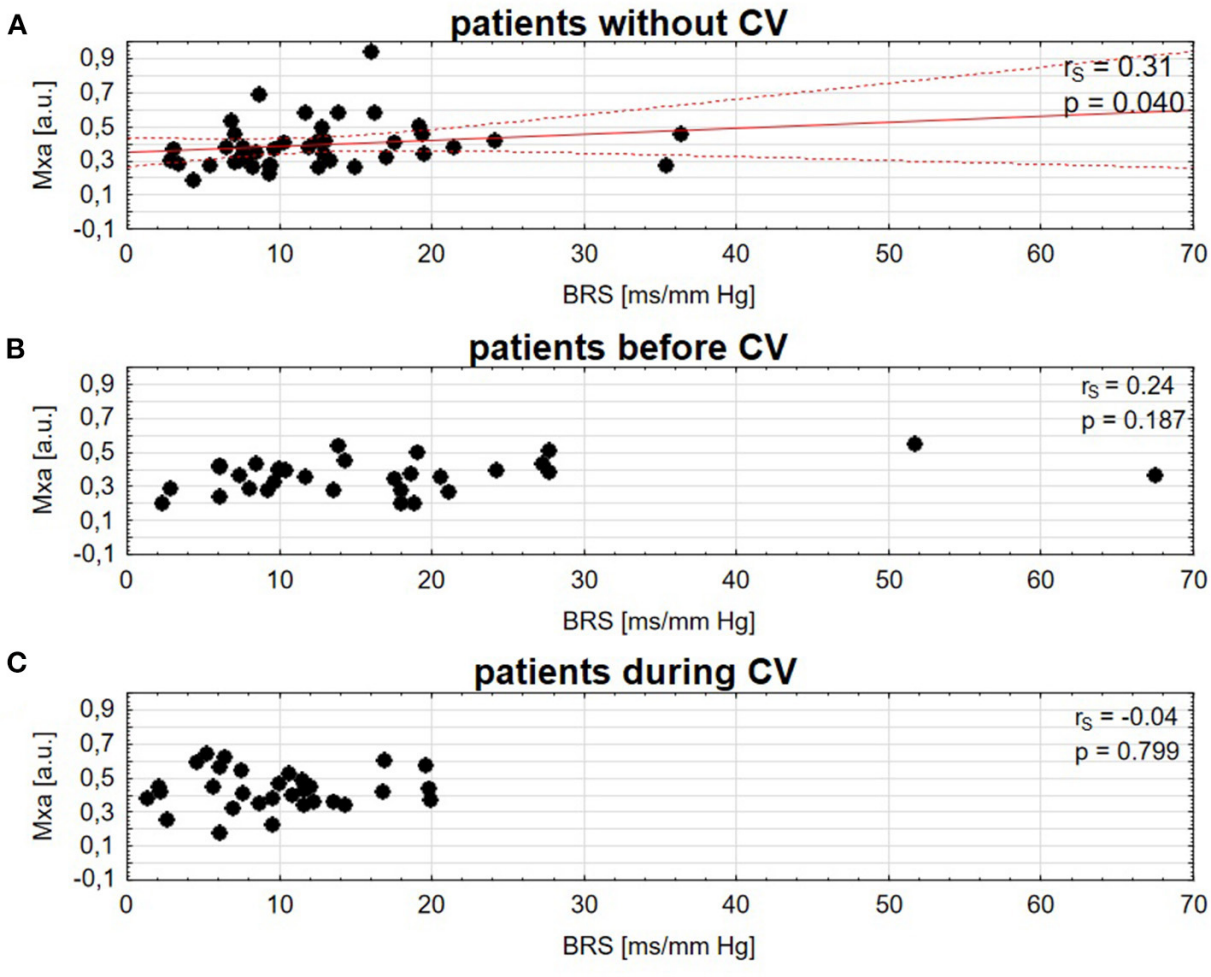
Spearman correlation of cerebral autoregulation (Mxa) and baroreflex sensitivity (BRS) in patients **(A)**, without cerebral vasospasm (CV), **(B)** before CV, **(C)** during CV. A solid red line is a linear regression and dashed lines represent 95% CI.

There was a significant and moderate correlation between Mxa and BRS in patients without CV: *r*_s_ = 0.31, *p* = 0.040 (higher—better BRS associated with higher—worse CA), Figure 3A. There was no significant correlation between Mxa and BRS in the CV group, neither before vasospasm (*r*_s_ = 0.24, *p* = 0.187, Figure 3B) nor during vasospasm (*r*_s_ = −0.04, *p* = 0.799, Figure 3C). There was no relationship between impairment of CA, BRS, and DCI.

### The Potential Impact of Treatment With Vasopressors

There were 26 (36%) patients who received vasopressors: 8 (19%) without CV, and 18 (58%) with CV. There was no significant difference for BRS between patients with vasopressors (*n* = 26) and patients without vasopressors (*n* = 47): *Z* = −0.81; *p* = 0.420. Moreover, in the CV group, BRS was significantly lower during CV compared with the period before CV both in patients with vasopressors (*Z* = 2.85; *p* = 0.004) as in patients without vasopressors (*Z* =2.97; *p* = 0.003). Loss of inverse correlation between BRS and Mxa before and during CV was found in the CV group, regardless of treatment with vasopressors. In the non-CV group, the inverse correlation between Mxa and BRS was not found when patients treated with vasopressors were excluded. However, the analysis of the impact of vasopressors on the relationship between BRS and CA could be biased by the limited number of observations in this subgroup.

### Time Trends of Physiological Metrics, Baroreflex Sensitivity and Cerebral Autoregulation in the CV and Non-CV Groups

**Figure 4:**
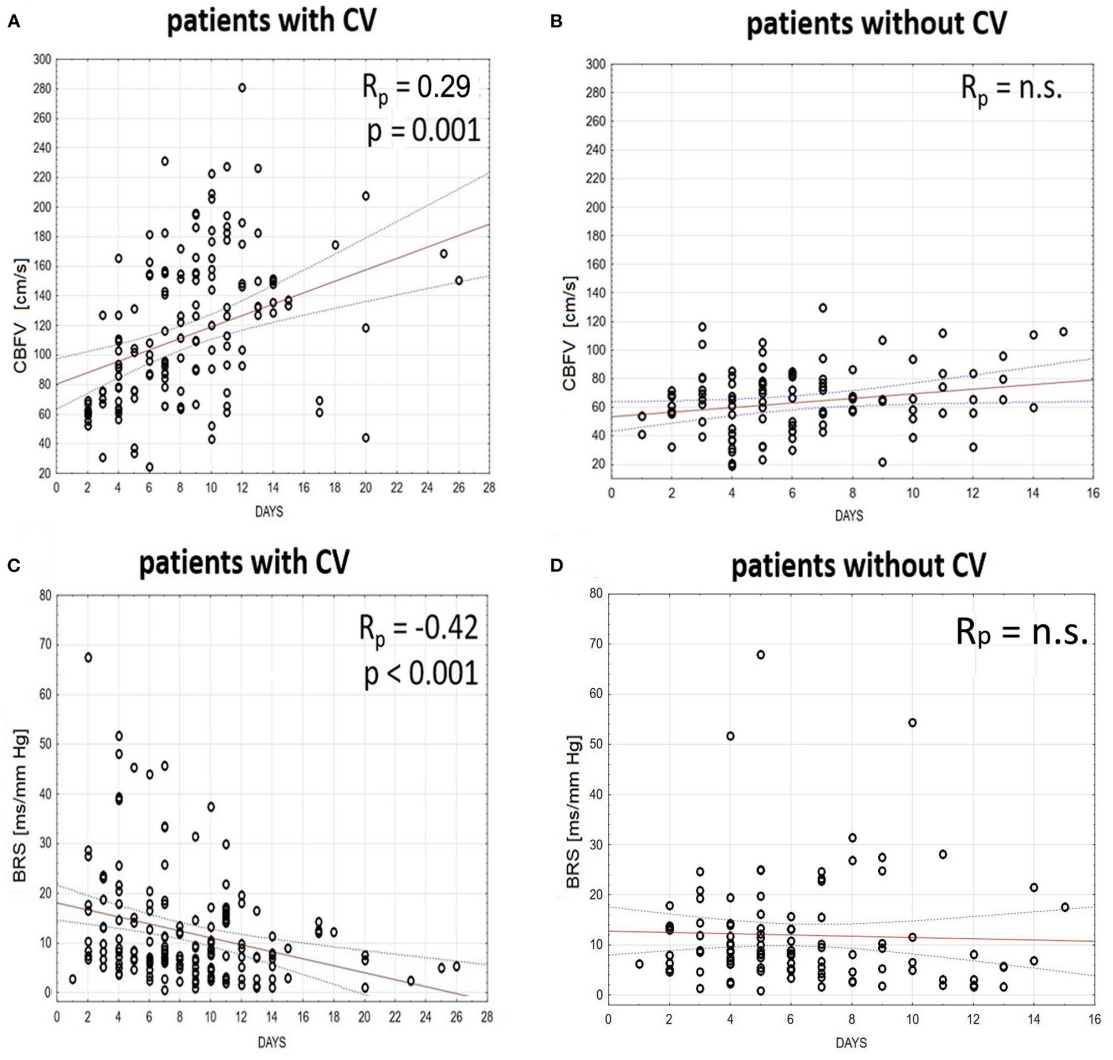
Time trends (solid red line) and the 95% CI (dashed line) of **(A,B)** cerebral blood flow velocity (CBFV) and **(C,D)** baroreflex sensitivity (BRS) in patients **(A,C)** with and **(B,D)** without cerebral vasospasm (CV) analyzed in the days that follow aneurysmal subarachnoid hemorrhage (aSAH). The relationship between days and physiological parameters was calculated using multiple linear regression analyses with subjects treated as categorical factors using dummy variables (with respect to the intersubject variability) and a partial coefficient (R_p_) between analyzed variables.

In patients with CV, multiple linear regression analysis showed a significant increase in ABP, HR, and CBFV in the days that followed aSAH—ABP: *R*_p_ = 0.31, *p* < 0.001; HR: *R*_p_ = 0.46, *p* < 0.001; CBFV: *R*_p_ = 0.29, *p* < 0.001. Furthermore, a moderate decrease in BRS was found in the days that followed aSAH in patients with CV: *R*_p_ = −0.42; *p* < 0.001 (Figure 4), but not in patients without CV.

## Discussion

Inverse correlation between BRS and CA was found to be lost in aSAH in patients with vasospasm both during vasospasm and during the period that preceded vasospasm. BRS decreased substantially in these patients during vasospasm as compared to the period before vasospasm. In patients without CV, a significant moderate correlation was found between BRS and CA, with worse BRS being associated with better CA, a finding previously termed as “inverse correlation” (7). Multiple linear regression analysis showed a significant relationship between the decrease in BRS, and the days elapsed from aSAH in the CV group.

Cerebral autoregulation regulates cerebral blood flow (CBF) in the distal arteries and stabilizes CBF in the face of changes in ABP mainly through myogenic and neurogenic mechanisms (18). CA is frequently impaired after aSAH (19, 20), even without any evidence of changes in flow velocity in the major arteries (21). It was found in this study that CA was getting worse during the CV. This observation is consistent with previous studies in patients with SAH, where Mxa was higher in patients with vasospasm compared to baseline (22, 23). It was shown that autoregulation is impaired in most patients with vasospasm of large cerebral arteries due to low-perfusion pressure distal to large arteries, and responsive maximal dilation of downstream vessels (23, 24).

Inverse correlation between CA and BRS was preserved in patients without CV. From a dynamic systems theory, the interaction between BRS and CA provides stable perfusion of the brain. The failure of both systems has previously been reported before syncope (25). The compensatory interaction between blood pressure regulation and cerebral perfusion pressure mechanisms was found in a previous study in the healthy young humans (7, 26). These studies have shown that impaired CA was counterbalanced with better BRS and *vice-versa*. The same observation has been found in patients with atherosclerotic stenosis or occlusion (7) and also in a cohort of patients with aSAH (6). Regulation of CBF is maintained through both arterial baroreflex control of blood pressure and CA operating through changes of cerebrovascular resistance. However, the relation between these two homeostatic mechanisms is still not clear. Rather than interpretation in a finalistic view, a mechanistic approach has been favored to explain the inverse correlation, linking better CA to enhanced cerebral vasomotor tone due to the increased sympathetic activity associated with diminished BRS (7). A finalist view, however, cannot be excluded and can be based on the theory of the physiological system complexity states that the structure is optimally configured to support its functions (27).

In this study, we found that inverse correlation between CA and BRS is lost in patients with aSAH, who developed CV. This finding could be the result of mechanical alterations caused by the local modifications in the cerebral vasomotor tone as a consequence of the degradation of hemoglobin after aSAH (28) that can potentially override the systemic input of the cardiovascular autonomic nervous system on CA. The potential consequences of the observed loss of inverse correlation between BRS and CA are not known. It might play a role in the occurrence of delayed ischemic deficit in a proportion of patients who develop CV after aSAH. However, we found that impaired BRS and CA occurred both in patients with DCI and non-DCI. Potentially improving both of these metrics should be targeted in post-SAH clinical management.

The findings on the relationship between the BRS–CA relationship and age are currently inconclusive. The correlation between pressure index reactivity (PRx) and BRS has been shown to be reciprocal in the elderly patients (>60 years) with traumatic brain injury (15). On the other hand, a study in the healthy volunteers (20–80 years) has shown that in older subjects BRS and CA were not correlated (29). Previous studies have shown that spontaneous BRS decreases significantly with age and that the loss of arterial distensibility with age would be the main mechanism responsible for the reduction in BRS in the older subjects. However, in our study, patients with CV were younger than patients without CV, so the decrease in BRS in that group of patients is not biased by age (30).

Another result from this study pertains to the behavior of BRS as a time-trend parameter, decreasing during the days that followed aSAH. BRS decreased significantly after aSAH and “stunned” values of BRS were observed in patients with CV almost three weeks after onset. In the non-CV group, a non-significant trend to a moderate decrease in BRS was also observed in the days following aSAH.

Baroreflex is central to the cardiovascular homeostasis maintained by the autonomic nervous system. An increase in sympathetic activity is associated with lower BRS (31). Autonomic nervous system impairment is common in patients with acute brain insult (32, 33). It can also occur in the subacute neurological diseases, such as Guillain–Barre syndrome (34). Thus, BRS impairment reflects a non-specific systemic impairment of the cardiovascular autonomic nervous system in the context of acute brain injury. Our study was not designed to assess the clinical significance of the loss of inverse correlation between CA and BRS during the CV. However, in case future studies confirm the loss of inverse correlation between BRS and CA in patients at risk of clinical deterioration after aSAH a comprehensive continuous real-time assessment of BRS along with CA could provide valuable information in patients with aSAH as both BRS and CA have been shown to be associated with outcome at 3 months (4, 5). In less acute pathological situations, medical therapy combined with baroreflex activation therapy (BAT) has been successful, such as in resistant hypertension (35) and heart failure with reduced ejection fraction (36). It is yet to demonstrate that BRS is a potential therapeutic target after an acute brain injury such as aSAH.

The cohort of patients utilized in our study has already been analyzed for assessment of prognosis in relation to BRS (5), and cerebral hemodynamic parameters (4, 37, 38). In this study, we aimed to assess integrative pathophysiology evaluating the interaction between the two main cardiovascular and cerebral homeostatic mechanisms that have been associated with prognosis after aSAH, e.g., BRS (5) and CA (4). The signal recordings analyzed in this study were routinely sampled at 200 Hz frequency and were appropriate for the calculation of xBRS in addition to CA assessment. The fact that signals have been recorded a few years ago have probably not affected the conclusions of this study. Concerning aSAH treatment, it has slightly changed from the guidelines applicable at the time of admission with the most significant change concerning the recommendation for systemic and cardiovascular monitoring to be applied (39).

### Limitations

This study has several limitations. First, a relatively limited number of patients were included. In addition, patients were not stratified according to the previous comorbidities. DSA, which is assumed to be the gold standard for detecting the cause of SAH and for the preoperative evaluation of aSAH, was not available in all the included cases. Therefore, in those patients, aSAH had been diagnosed by CT angiography. A small proportion of patients had conservative treatment because of the poor grade SAH. A potentially confounding factor was the younger age of the patients with CV compared to the patients without CV in our study. This could be due to a bias related to the fact that in our study we used the same threshold for velocities to define vasospasm both in the young and old patients, although there is evidence that in older patients, CV is associated with lower velocities (40). However, there are no recommendations to diagnose CV in older patients based on different thresholds. As a consequence of the classic choice we made for similar thresholds for velocities to diagnose CV in younger and older patients, CV could have been underdiagnosed in the older patients. Another potentially confounding factor was a higher ABP in the CV group, which is the expected result of the applied therapeutics. However, it is unlikely that this result has biased the BRS assessment. Indeed, although resetting BRS to adapt to higher ABP values is known to occur in chronically hypertensive patients (41), there is no evidence that a short-term increase in ABP modifies BRS. Our study included mechanically ventilated patients, which could be another potential confounding factor. A previous study has shown that mechanical ventilation can attenuate respiratory arrhythmia and alter BRS (42). However, in this study, there was no significant difference in BRS between mechanically ventilated (*n* = 24) and non-mechanically ventilated patients (*n* = 49). Also, we found that BRS was significantly lower during CV compared to before CV both in patients with CV and mechanical ventilation (*p* = 0.016) and in patients with CV and without mechanical ventilation (*p* = 0.001). Furthermore, the loss of inverse correlation between Mxa and BRS was observed before vasospasm and during vasospasm in patients with CV regardless of mechanical ventilation. Thus, it seems unlikely that the correlation between BRS and CA was biased by mechanical ventilation. This study included sedated and mechanically ventilated patients in whom the main sedation drugs used were propofol and fentanyl. Propofol administration has been reported to reduce sympathetic autonomic outflow and decrease ABP due to its vasodilator effect (43, 44). However, propofol was shown to not significantly change the average HR, based on which BRS is measured (45, 46). As for fentanyl, it was reported that its effect on the autonomic nervous system is highly dose dependent. Our study population was homogeneous in terms of medical procedures and the use of drugs. However, patients have received individually titrated doses of vasopressors, which may alter BRS. Although in our study there was no significant difference in BRS between patients with vasopressors and patients without vasopressors, more studies are needed to assess the impact of vasopressors on the relationship between BRS and CA in patients with aSAH.

## Conclusion

Inverse correlation between BRS and CA was lost in patients with CV following aSAH. Furthermore, BRS significantly decreased during the days that followed aSAH in patients who developed CV. These findings may have an impact on the prognosis of these patients and need to be investigated in larger multicentric studies.

## Data Availability Statement

The raw data supporting the conclusions of this article will be made available by the authors, without undue reservation.

## Ethics Statement

The studies involving human participants were reviewed and approved by research Ethics Committee at Addenbrooke's Hospital (Protocol 29 LREC: 97/291). The patients/participants or their next-of-kin provided written informed consent to participate in this study.

## Author Contributions

Study concept and design, drafting of the manuscript, and literature search was done by AU, NN, MK, and MB. Figure acquisition was perfomed by AU and NN. Critical revisions were done by AU, NN, MK, PS, MC, KB, MS, and MB. MC, PS, MS, and MB supervised the article. All authors contributed to the article and approved the submitted version.

## Funding

This work was supported by (National Science Centre, Poland, under the MINIATURA 5 grant, Nr DEC-2021/05/X/ST7/00454 and Foundation for Polish Science FNP) to AU, (Polish National Agency for Academic Exchange under the International Academic Partnerships program) to MK, and (NIHR, Biomedical Research Centre Cambridge, United Kingdom) to MC.

## Conflict of Interest

MC and PS are authors of ICM+ software. They have a financial interest in a part of the licensing fee for ICM + distributed by Cambridge Enterprise Ltd., UK. The remaining authors declare that the research was conducted in the absence of any commercial or financial relationships that could be construed as a potential conflict of interest.

## Publisher's Note

All claims expressed in this article are solely those of the authors and do not necessarily represent those of their affiliated organizations, or those of the publisher, the editors and the reviewers. Any product that may be evaluated in this article, or claim that may be made by its manufacturer, is not guaranteed or endorsed by the publisher.
